# Pigeon paramyxovirus type 1 from a fatal human case induces pneumonia in experimentally infected cynomolgus macaques (*Macaca fascicularis*)

**DOI:** 10.1186/s13567-017-0486-6

**Published:** 2017-11-21

**Authors:** Thijs Kuiken, Pascal Buijs, Peter van Run, Geert van Amerongen, Marion Koopmans, Bernadette van den Hoogen

**Affiliations:** 1 Department of Viroscience, Erasmus University Medical Centre Rotterdam, Rotterdam, The Netherlands; 2 Present Address: Department of Surgery, Elisabeth-TweeSteden Hospital, Tilburg, The Netherlands; 3 Centre for Infectious Disease Control, National Institute for Public Health and the Environment, Bilthoven, The Netherlands

## Abstract

Although avian paramyxovirus type 1 is known to cause mild transient conjunctivitis in human beings, there are two recent reports of fatal respiratory disease in immunocompromised human patients infected with the pigeon lineage of the virus (PPMV-1). In order to evaluate the potential of PPMV-1 to cause respiratory tract disease, we inoculated a PPMV-1 isolate (hPPMV-1/Netherlands/579/2003) from an immunocompromised human patient into three healthy cynomolgus macaques (*Macaca fascicularis*) and examined them by clinical, virological, and pathological assays. In all three macaques, PPMV-1 replication was restricted to the respiratory tract and caused pulmonary consolidation affecting up to 30% of the lung surface. Both alveolar and bronchiolar epithelial cells expressed viral antigen, which co-localized with areas of diffuse alveolar damage. The results of this study demonstrate that PPMV-1 is a primary respiratory pathogen in cynomolgus macaques, and support the conclusion that PPMV-1 may cause fatal respiratory disease in immunocompromised human patients.

## Introduction

Newcastle disease virus, or avian paramyxovirus type 1 (APMV-1), is classified in the genus *Avulavirus*, family Paramyxoviridae [1]. Genetic and antigenic analyses have demonstrated that APMV-1 strains cluster in 10 different genotypes, with different sublineages [2]. Based on their difference in reactivity in hemagglutination assays, and their host specificity for columbiform species, all pigeon paramyxoviruses (PPMV-1) cluster in lineage VIb/1 of genotype VI APMV-1 strains [2–5].

APMV-1 infection is known to cause mild disease in human beings. Most commonly, human infection is associated with conjunctivitis, which resolves within 1 week. Less often, it is associated with transient fever or chills [6–9]. So far, only two fatal cases of human infection have been reported, one in the USA [10] and the other in the Netherlands [11] (unpublished observations). Both patients had an immunosuppressive condition as a comorbidity and developed severe pneumonia. Both virus isolates from the patients were typed as PPMV-1. PPMV-1 was first detected in Southeastern Europe in the 1980s and became established in pigeon populations across Europe. From there, PPMV-1 has spread and continues to spread worldwide. PPMV-1 is now established in both domestic and wild pigeons and doves in Europe and North America, from where it can often spread to poultry, with outbreaks reported [2, 12].

Strains of APMV-1 and PPMV-1 can be categorized into three different groups based on the severity of disease they cause in birds: lentogenic (avirulent), mesogenic (intermediate virulent) and velogenic (virulent). These differences in virulence can be explained mostly by differences in the cleavage site of the fusion (F) protein, which is synthesized in an inactive form (F0) and needs to be proteolytically cleaved by host cellular proteases into active F1 and F2 forms in order to become functional. Activation of the F0 protein will result in fusogenic activity promoting virus-to-cell fusion and cell-to-cell fusion. Lentogenic viruses have a monobasic cleavage site, which can only be cleaved by trypsin-like proteases found in the respiratory and digestive tracts of birds. More virulent strains have a polybasic cleavage site, which can also be cleaved by furin-like proteases, found more abundantly in most organs and explaining why virulent strains can replicate systemically in birds [13].

APMV-1 strains have been tested in different animal models to evaluate their virulence for mammals. Inoculation of lentogenic, mesogenic, and velogenic strains of APMV-1 induced conjunctivitis in rhesus macaques (*Macaca mulatta*) and rabbits, but only after mild mechanical abrasion of the conjunctival epithelium [14]. Intranasal combined with intratracheal inoculation of a mesogenic strain of APMV-1 (Beaudette C) into African green monkeys (*Cercopithecus aethiops*) resulted in a low level of replication at scattered locations in the lung, without overt disease signs: pathological changes were not evaluated [15]. Intranasal inoculation of a lentogenic strain of APMV-1 (La Sota) into BALB/c mice [16] or Syrian golden hamsters [17] resulted in body weight loss and, at 3 days post inoculation (dpi), necrotizing broncho-interstitial pneumonia associated with virus antigen expression in bronchial epithelium. However, the virulence of PPMV-1 in laboratory mammals, as a model for human disease, has not been evaluated.

We used the PPMV-1 isolate from a fatal human case (hPPMV-1/Netherlands/579/2003) to investigate the potential of this virus to cause respiratory tract disease in a non-human primate. To this end, three cynomolgus macaques (*Macaca fascicularis*) were inoculated and pathological, immunohistochemical and virological analyses were performed at 3 dpi. We demonstrate that PPMV-1 can cause severe virus-associated pneumonia in a non-human primate model.

## Materials and methods

### Virus preparation

The hPPMV-1/Netherlands/579/2003 isolate was obtained from an adult female patient from the Netherlands with multiple myeloma, who died from respiratory failure after receiving allogenic bone marrow transplantation and immunosuppressive treatment [11] (unpublished observations). The virus isolate was passaged twice on human rhabdomyosarcoma cells and once on Vero cells. Prior to inoculation, the virus isolate was cultured in 10-day-old specific-pathogen-free embryonated chicken eggs using standard techniques, and harvested at 2 dpi. The virus stock was titrated by end-point dilution assay in Vero clone 118 cells. To read out infection, cells were stained with chicken polyclonal anti-APMV-1 antibody and rabbit FITC-labeled anti-chicken IgG antibody (1:2000 and 1:1000 dilution respectively; both from Abcam, Cambridge, UK) 72 h after inoculation. Viral titers were calculated using the method of Reed and Muench [18]. The infectious virus titer of this stock was 5 × 10^8^ median tissue culture infective dose (TCID_50_) per mL.

### Experimental protocol

Three male cynomolgus macaques, 3.5 years old, were colony bred and had been maintained in group housing, where they were screened annually—and tested negative—for the following infections: simian virus 40, polyomavirus, *Mycobacterium tuberculosis*, measles virus, mumps virus, simian immunodeficiency virus, simian retrovirus type D, and simian T cell leukemia virus. Prior to infection, they were examined clinically and determined as healthy by a registered veterinarian. One month before the start of the experiment, a Data Storage Tag centi-Temperature probe (Star-Oddi, Brussels, Belgium) was implanted intraperitoneally, set to register temperature every 10 min. One week before inoculation, the macaques were placed together in a negatively pressurized, HEPA-filtered isolator cage. They were provided with commercial food pellets and water ad libitum. The macaques were inoculated with 1.0 × 10^8^ TCID_50_ of hPPMV-1/Netherlands/579/2003, which was suspended in 4.5 mL of phosphate-buffered saline (PBS). Approximately 3 mL was applied intratracheally by use of a catheter, 0.5 mL in each nare, and 0.25 mL on each of the conjunctivae. The macaques were observed daily for the occurrence of malaise, coughing, exudate from the eyes or nose, forced respiration, and any other signs of illness. The macaques were euthanized at 3 dpi by exsanguination under ketamine and medetomidine hydrochloride anesthesia. Just before infection and daily until euthanasia, the macaques were anesthetized with ketamine and medetomidine hydrochloride, and pharyngeal, nasal, ocular, and rectal swabs were collected in 1 mL transport medium [19] and stored at −70 °C until quantitative RT-PCR (qRT-PCR) and/or virus isolation. In addition, 4 mL blood was taken from an inguinal vein at each of these time points and collected in Vacuette Z Serum Sep Clot Activator tubes (Greiner Bio One, Alphen aan den Rijn, The Netherlands).

### Clinical biochemistry

Clotted blood samples were centrifuged and 100 µL separated serum was assayed with Piccolo BioChemistry Panel Plus Reagent Discs (Abaxis, Darmstadt, Germany), which were processed using a Piccolo Xpress chemistry analyzer (Abaxis) following the manufacturer’s instructions. Measurements were obtained for glucose, blood urea nitrogen, creatinine, calcium, albumin, total protein, alanine aminotransferase, aspartate aminotransferase, alkaline phosphatase, γ-glutamyl transpeptidase, amylase and C-reactive protein. Reference values (if indicated) were obtained from the supplemental data of a publication by Xie et al. [20].

### Autopsy and tissue sampling

Autopsies were carried out according to a standard protocol. Organs were examined for lesions and sampled for laboratory analyses. For histopathology and immunohistochemistry, samples of adrenal gland, brain stem, cerebellum, cerebrum, conjunctiva, ethmoid bone, eye, eyelid, heart (left and right ventricle), kidney, large intestine, lung (left and right; upper, middle and lower lobes), liver, mesenteric lymph nodes, nasal conchae, pancreas, small intestine, spleen, tonsil and tracheo-bronchial lymph nodes were collected in 10% neutral-buffered formalin (lungs after inflation with formalin) and allowed to fix for 1 week. For qRT-PCR, broncho-alveolar lavage (BAL) was performed by direct infusion of PBS into the right main bronchus. Recovered BAL fluid was centrifuged and the cellular pellet resuspended in TRIzol and stored at −80 °C until further analysis. In addition, samples of adrenal gland, brain, cerebrospinal fluid, conjunctivae, heart, kidney, mesenteric lymph nodes, lung (right upper and lower lobes), large intestine, liver, nasal conchae, nasal septum, pancreas, primary bronchus, small intestine, spleen, trachea, and tracheo-bronchial lymph nodes were collected in RNAlater (Life Technologies, Bleiswijk, The Netherlands) and stored at −80 °C until further analysis. For virus isolation, samples from the same tissues as for qRT-PCR were stored without additives at −80 °C until further analysis.

### Histopathology

Samples for histopathological analysis were embedded in paraffin, sectioned at 3 µm, and stained with hematoxylin and eosin (H&E) for examination by light microscopy. Tissue sections of a clinically healthy juvenile cynomolgus macaque that had not been infected with PPMV-1 were used as a negative control.

### Immunohistochemistry

Formalin-fixed, paraffin-embedded, 3-µm-thick sections of the same tissues examined histopathologically were stained using an immunoperoxidase method. Tissue sections were mounted on coated slides (Klinipath, Duiven, The Netherlands), deparaffinized and rehydrated. Endogenous peroxidase was blocked by incubating sections in 3% H_2_O_2_ in PBS for 10 min at room temperature (RT). Antigen was retrieved by Tris–EDTA buffer (pH 9) for 15 min. Sections were subsequently washed with PBS containing 0.05% Tween 20 (Fluka, Chemie AG, Buchs, Switzerland) and incubated in PBS with 0.1% BSA (Aurion, Wageningen, The Netherlands) for 10 min at RT. After this, sections were incubated in PBS with 0.1% BSA with a monoclonal mouse antibody IgG2a to APMV-1 (dilution 1:100, MAb 6H12, specific to ribonucleoprotein; La Sota strain, Hytest Ltd, Turku, Finland) or with a negative control isotype mouse monoclonal antibody (dilution 1:100, MAb 003, R&D System, Minneapolis, USA) for 1 h at RT. After washing, sections were incubated with goat anti-mouse antibody (dilution 1:400, Southern Biotech, Birmingham, AL, USA) labeled with horseradish peroxidase (HRP) for 1 h at RT. HRP activity was revealed by incubating the sections in 3-amino-9-ethylcarbazole (Sigma Chemical Co., St. Louis, USA) in *N*,*N*-dimethylformamide (Sigma Chemical Co.) solution for 10 min at RT, resulting in a red precipitate. Sections were counterstained with hematoxylin. Brain tissue sections from a cormorant (*Phalacrocorax auritus*) known to be infected with APMV-1 were used as a positive control. Tissue sections of a clinically healthy juvenile cynomolgus macaque that had not been infected with PPMV-1 were used as a negative control.

### RNA isolation and qRT-PCR assay

BAL samples stored in TRIzol were processed according to the manufacturer’s instructions to isolate RNA. Tissue samples stored in RNAlater were weighed, thawed, transferred to tubes containing a quarter-inch-diameter ceramic sphere in virus transport medium, and homogenized using a FastPrep 24 tissue homogenizer (MP Biomedicals, Eindhoven, The Netherlands). The homogenates were centrifuged, and the cleared supernatants were used for RNA isolation. 200 µL of the cleared supernatant of tissue samples, as well as 200 µL of other samples (transport medium of swabs, plasma) were combined with 300 µL lysis buffer of the Total Nucleic Acid Isolation kit (Roche, Woerden, The Netherlands) and RNA was isolated in a volume of 50 µL using a MagNA Pure LC machine (Roche) following the manufacturer’s instructions.

NDV-specific qRT-PCR was performed on 5 µL (TRIzol samples) or 19.5 µL (MagNA Pure samples) RNA in an ABI PRISM 7000 Sequence Detection System using TaqMan Fast Virus 1-Step Master Mix (both from Life Technologies) in a total volume of 30 µL, using primers as described by Wise et al. [21]. The RT step was 5 min at 50 °C, followed by 95 °C for 20 s. Cycling consisted of 45 cycles of 3 s denaturation at 95 °C, 5 s annealing at 54 °C and 31 s extension at 60 °C.

### Virus isolation

200 µL transport medium from collected swabs or 200 µL supernatant from homogenized tissue samples was injected in duplicate into 10-day-old specific-pathogen-free embryonated chicken eggs. After 2 days, allantoic fluid was harvested and tested for presence of virus by hemagglutination assay.

## Results

### Clinical findings of experimentally infected macaques

The body temperature of macaque #2 increased to 39.5 °C at 0.5 dpi, compared to 38.5 °C for macaque #1 and 38.0 °C for macaque #3 at that time point. Otherwise, no clinical signs were seen. Clinical chemical changes in the plasma were limited to creatinine and C-reactive protein (Table 1). The creatinine concentration of macaque #2 was slightly elevated at 1 dpi, but returned to normal concentrations at 2 and 3 dpi. The creatinine concentrations of the other macaques remained within the normal range. The C-reactive protein concentrations in all macaques were increased at 1 dpi, and returned below the level of detection at 2 and 3 dpi.

**Table 1:** **Temporal course in clinical chemical values in the plasma of cynomolgus macaques inoculated with hPPMV-1/Netherlands/579/2003**

Analyte	Unit	Normal range	Macaque #1				Macaque #2				Macaque #3			
			D0	D1	D2	D3	D0	D1	D2	D3	D0	D1	D2	D3
Glucose	mmol/L	2.16–7.92	4.6	5.7	3.5	4.5	5.5	6.8	5.8	7.9	4.9	6.1	4.8	7.2
Blood urea nitrogen	mmol/L	4.36–9.28	4.9	7.6	5.6	5.2	5.1	5.9	5.3	5.8	5.8	7.2	6.7	6.6
Creatinine	µmol/L	30.57–72.37	57	60	49	70	59	93	65	64	49	68	53	51
Uric acid	µmol/L	na	<18	<18	<18	<18	<18	<18	<18	<18	<18	<18	<18	<18
Calcium	mmol/L	2.32–2.96	2.54	2.43	2.55	2.39	2.53	2.43	2.51	2.38	2.53	2.47	2.58	2.47
Albumin	g/L	28.31–50.79	27	23	25	24	31	30	29	28	32	29	31	31
Total protein	g/L	61.91–88.11	65	62	61	61	65	66	64	63	67	64	67	66
Alanine aminotransferase	U/L	2.95–87.03	58	60	77	82	134	103	87	84	42	52	56	70
Aspartate aminotransferase	U/L	24.30–69.90	51	62	82	148	166	61	90	127	43	56	80	106
Alkaline phosphatase	U/L	121.22–727.22	175	159	158	149	335	307	310	322	374	334	334	318
Gamma glutamyltransferase	U/L	14.61–62.53	75	62	66	63	24	25	25	24	52	46	50	47
Amylase	U/L	na	459	321	661	471	268	173	262	217	378	308	378	347
C-reactive protein	mg/L	na	<5	10.1	<5	<5	<5	98.4	<5	<5	<5	7	<5	<5

na: not available.

### Gross pathology

**Figure 1:**
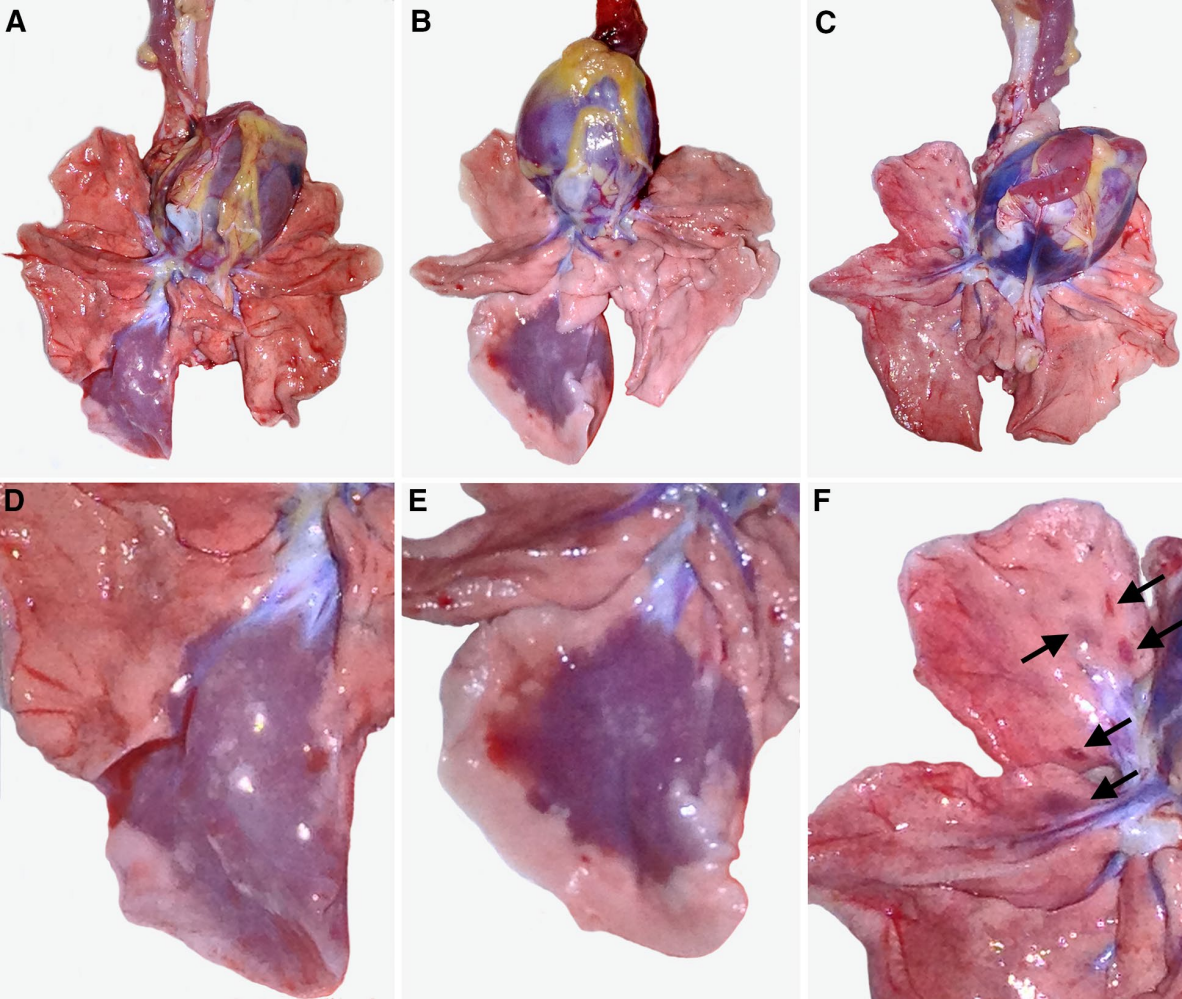
**Gross pathology of experimental hPPMV-1/Netherlands/579/2003 infection in cynomolgus macaques. A**–**C** Overview of the ventral aspect of the lungs. **D**, **E** Close-up of the right lower lung lobe. **F** Close-up of the right upper and middle lung lobes. There is a locally extensive area of consolidation in the right lower lung lobe of macaques #1 (**A**, **D**) and #2 (**B**, **E**), likely due to a substantial part of the inoculum flowing into this lobe. There are multiple areas of consolidation (arrows) in the right upper and middle lung lobes of macaque #3 (**C**, **F**).

All three macaques had locally extensive or multifocal pulmonary lesions, which were red–purple, slightly raised, and firmer than normal (Figure 1). The estimated volume of affected lung tissue differed per macaque: 30% (macaque #1), 25% (macaque #2), and 1% (macaque #3). The only gross lesions outside the respiratory tract were in lymphoid organs. All three macaques had enlarged tracheo-bronchial lymph nodes and prominent white pulp of the spleen, while macaque #1 also had enlarged tonsils and mandibular lymph nodes, and multiple red areas in mesenteric and pancreatic lymph nodes.

### Histopathology

**Figure 2:**
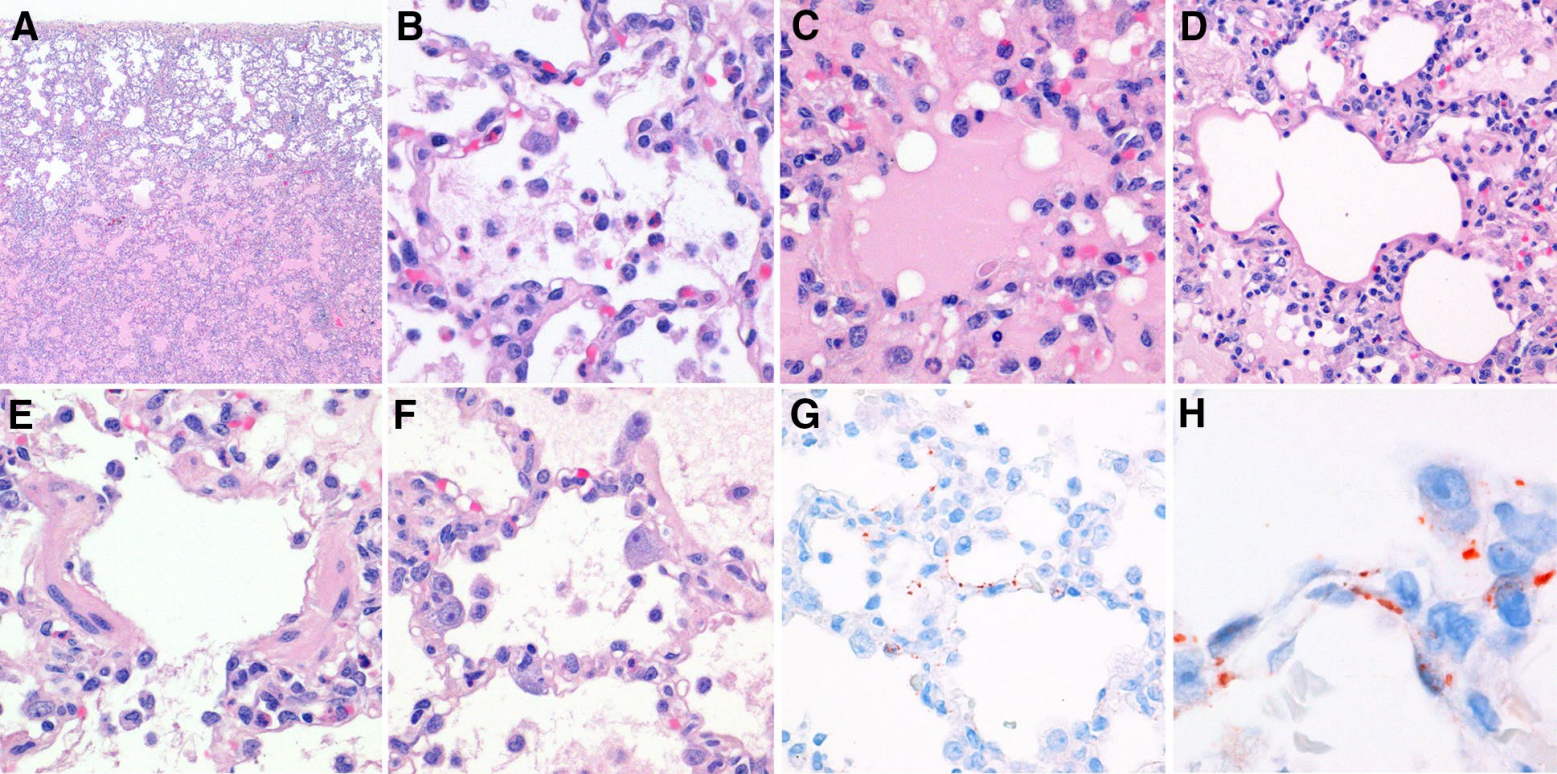
**Pulmonary histopathology and immunohistochemistry of experimental hPPMV-1/Netherlands/579/2003-infection in cynomolgus macaques. A** Pulmonary section of macaque #1. Diffuse alveolar damage. There is edema fluid and inflammatory cells in the alveolar lumina (H&E. Original magnification ×2). **B** Pulmonary section of macaque #1. Diffuse alveolar damage. There are macrophages, lymphocytes, neutrophils, and rare eosinophils, mixed with fibrin strands, in the alveolar lumen (H&E. Original magnification ×40). **C** Pulmonary section of macaque #1. Diffuse alveolar damage. In addition to inflammatory cells, there is abundant edema fluid in the alveolar lumen (H&E. Original magnification ×40). **D** Pulmonary section of macaque #1. Diffuse alveolar damage. Hyaline membranes lining the alveolar walls (H&E. Original magnification ×20). **E** Pulmonary section of macaque #1. Diffuse alveolar damage. There is loss of epithelial cells from the bronchiolar wall (H&E. Original magnification ×40). **F** Pulmonary section of macaque #2. Diffuse alveolar damage. Hypertrophy of type II pneumocytes lining the alveolar wall (H&E. Original magnification ×40). **G** Pulmonary section of macaque #2. Diffuse alveolar damage. Expression of PPMV-1 in epithelial cells of the alveoli (immunoperoxidase stain for PPMV-1. Original magnification ×40). **H** Pulmonary section of macaque #1. Diffuse alveolar damage. Expression of PPMV-1 in type I pneumocytes and type II pneumocytes (immunoperoxidase stain for PPMV-1. Original magnification ×100).

In the lung, all three macaques had multiple or coalescing foci of inflammation and necrosis that centered on the bronchioles (Figure 2). In these foci, the alveolar and bronchiolar lumina were filled with macrophages, lymphocytes, neutrophils, and rare eosinophils, mixed with edema fluid, fibrin, erythrocytes, and cellular debris. Alveolar and bronchiolar epithelia had evidence of both necrosis (denuded basement membranes and attenuated bronchiolar epithelial cells) and regeneration (type II pneumocyte hypertrophy and hyperplasia). The alveolar and bronchiolar walls were moderately thickened by the presence of mononuclear cells, neutrophils, and edema fluid. Some alveolar walls were lined by hyaline membranes. The bronchi were only mildly affected. There was moderate accumulation of lymphocytes around branches of both pulmonary arteries and pulmonary veins, and mild infiltration of mononuclear cells and neutrophils in pulmonary vein walls. The pleura overlying the inflammatory foci was infiltrated with mononuclear cells, neutrophils, and eosinophils.

**Figure 3:**
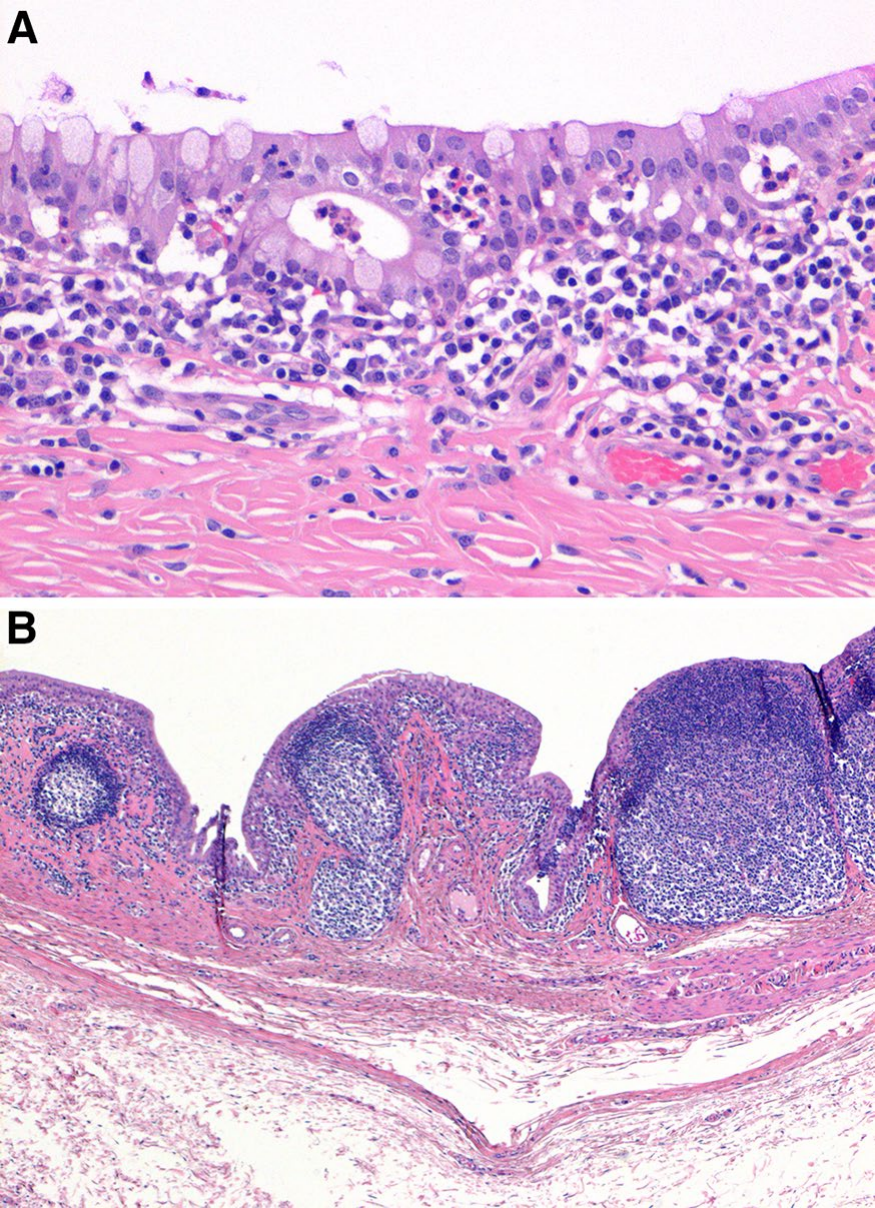
**Conjunctival histopathology of experimental hPPMV-1/Netherlands/579/2003 infection in cynomolgus macaques. A** Conjunctival section of macaque #3. Conjunctivitis. There are neutrophils in the subconjunctival connective tissue and conjunctiva (H&E. Original magnification ×20). **B** Conjunctival section of macaque #3. Conjunctivitis. Enlarged lymphoid follicles in the subconjunctival connective tissue (H&E. Original magnification ×2).

The lymph nodes and spleen had benign lymphoid hyperplasia, characterized by an increased number of lymphocytes and expansion of germinal centers. In addition, the medullary sinuses in the pancreatic and mesenteric lymph nodes of macaque #1 contained many erythrocytes; furthermore, the colon of this macaque had multiple small hemorrhages in the lamina propria. Macaques #1 and 2 had a mild superficial tonsillitis, characterized by the presence of neutrophils and bacterial colonies in the lumen and epithelium of the tonsillar crypt. Macaque #3 had a mild superficial conjunctivitis, characterized by the presence of neutrophils, lymphocytes, plasma cells, and expansile lymphoid follicles in the subconjunctival connective tissue, and migration of neutrophils through the conjunctiva into the lumen (Figure 3).

### Immunohistochemistry

Expression of PPMV-1 was visible as red–brown granular cytoplasmic staining in alveolar and bronchiolar epithelial cells in affected areas of the lung in all three macaques (Figure 2). Neither unaffected areas of the respiratory tract nor extra-respiratory organs showed PPMV-1 expression. PPMV-1 expression was present in the positive control tissue and absent in the isotype control and negative control tissues.

### qRT-PCR and virus isolation

**Figure 4:**
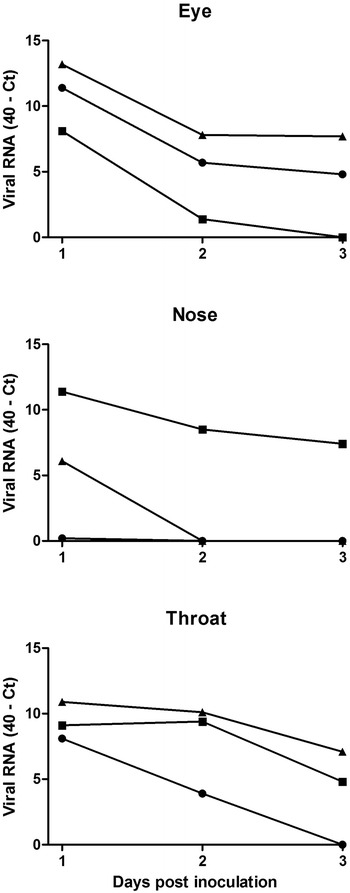
**Detection of hPPMV-1/Netherlands/579/2003 by qRT-PCR in experimentally infected cynomolgus macaques.** Circle: macaque #1, square: macaque #2, triangle: macaque #3.

By qRT-PCR, the highest levels of PPMV-1 RNA were detected in eye, nose, and throat swabs of all three macaques at 1 dpi (Figure 4). The amount of RNA declined on subsequent days, but RNA was still detectable at 3 dpi in 1 (nose swab) or 2 out of three macaques (eye and throat swabs). Virus isolation conducted with RNA-positive swabs demonstrated the presence of infectious virus only at 1 dpi in the throat swabs of macaques #2 (titer: 1.0 × 10^1^ TCID_50_/mL) and #3 (2.3 × 10^2^ TCID_50_/mL) and the eye swab of macaque #3 (7.4 × 10^2^ TCID_50_/mL).

At autopsy, PPMV-1 was detected by qRT-PCR and virus isolation in the lung of all three macaques (Table 2). PPMV-1 also was detected by qRT-PCR but not by virus isolation in other respiratory tract tissues: nasal turbinate, trachea and bronchus. Except for tracheo-bronchial lymph node, which was positive by qRT-PCR but not by virus isolation, all extra-respiratory organs were negative for PPMV-1 by qRT-PCR and virus isolation.

**Table 2:** **Detection of hPPMV-1/Netherlands/579/2003 by qRT-PCR (threshold cycle value) and virus isolation (TCID**_**50**_ **/mL) in respiratory tract organs and tracheo-bronchial lymph nodes of cynomolgus macaques at 3 dpi**

Macaque no.	Nasal concha		Trachea		Bronchus		Upper lung lobe		Lower lung lobe		Broncho-alveolar lavage fluid		Tracheo-bronchial lymph node	
	qRT-PCR	VI	qRT-PCR	VI	qRT-PCR	VI	qRT-PCR	VI	qRT-PCR	VI	qRT-PCR	VI	qRT-PCR	VI
1	−	nd	34.9	–	35.8	–	–	nd	29.7	1.2 × 10^3^	25.1	nd	–	nd
2	36.4	–	37.0	–	37.3	–	38.8	1.6 × 10^1^	27.2	8.4 × 10^1^	22.8	nd	–	nd
3	32.2	–	38.7	–	–	nd	29.7	–	32.2	1.3 × 10^3^	23.3	nd	34.2	–

Adrenal gland, brain, cerebrospinal fluid, conjunctiva, heart, kidney, mesenteric lymph node, large intestine, liver, nasal septum, pancreas, small intestine, and spleen tested negative by qRT-PCR for PPMV-1.

VI: virus isolation; −: negative; nd: not done.

## Discussion

This experimental infection shows that a PPMV-1 isolate from a fatal human case can cause severe pneumonia in a non-human primate model. Histological lesions in PPMV-1-infected macaques consisted of diffuse alveolar damage, affecting up to 30% of the lungs. The co-localization of these lesions with specific expression of PPMV-1 antigen, together with the re-isolation of PPMV-1 from lung samples at autopsy, support our conclusion that PPMV-1 infection was the cause of pneumonia.

These findings largely correspond with the presentation of infection with PPMV-1 in two human fatal cases from New York [10] and The Netherlands [11] (unpublished observations). Both human patients also developed pneumonia. The histopathological characterization of the pneumonia in our macaques was nearly identical to that in the New York and Dutch patients. The cell tropism also appeared to be the same in our macaques (pneumocytes and bronchioloar epithelial cells) and the New York and Dutch patients, where cells expressing PPMV-1 antigen resembled sloughed pneumocytes. This sloughing may have been due to a prolonged interval between death and autopsy; it also would preclude definite differentiation between alveoli and adjacent bronchioles as the source of the sloughed epithelial cells. While it was suggested that the New York and Dutch patients had systemic infection based on PPMV-1-positive urine and stool samples (New York patient) and PPMV-1-positive extra-respiratory tissues (Dutch patient), we did not detect PPMV-1 in any extra-respiratory tissues—including kidney, small and large intestines, pancreas and liver—of our macaques, either by qRT-PCR, virus isolation, or immunohistochemistry. However, it cannot be excluded that under conditions of immunosuppression, the virus would be able to spread systemically in macaques.

The results of this study, together with those of two fatal human cases of PPMV-1 infection, indicate that some PPMV-1 can cause severe pneumonia in people and macaques under certain conditions. These conditions include immunosuppression, intratracheal route of infection, and high viral dose. It remains to be determined whether PPMV-1, and particularly these isolates from fatal human cases, are more virulent for people than other APMV-1 strains. APMV-1 is able to selectively replicate in cancer cells and therefore is a potential therapeutic agent in cancer therapy. During application of different APMV-1 strains by different routes—locally, subcutaneously, intravenously, by inhalation—in clinical trials involving thousands of people, severe adverse effects have not been recorded [22]. Also, preclinical studies employing APMV-1 as a vaccine vector by different routes, or employing APMV-1 as a high-dose intravenous cytolytic agent, all demonstrated that APMV-1 is not pathogenic in non-human primates [15, 23–28]. The virulence of PPMV-1—a pigeon-specific lineage of APMV-1—for humans and macaques suggests that the strain used should be carefully selected.
